# Of Jazz and Meal Preparation

**DOI:** 10.1007/s11606-025-09545-z

**Published:** 2025-04-24

**Authors:** Anna H. Gallion, Michelle Izmaylov

**Affiliations:** 1 Vanderbilt University Medical Center, Nashville, TN USA; 2 Vanderbilt University School of Medicine, Nashville, TN USA; 3 Veterans Affairs Tennessee Valley Healthcare System, Nashville, TN USA; 4 Vanderbilt University School of Nursing, Nashville, TN USA

We think that we learn much about our patients when they are tethered by IVs to their hospital rooms, their clothes swapped out for hospital gowns, the food on their trays brought from the cafeteria. We check on patients with the same hospital gowns, patients with the same food on their trays, patients in the same hospital rooms, and patients labeled by their diagnoses when we speak of them on rounds. In this environment, we think we come to learn about our patients as people.

I think I know the man that I admit for inpatient care in the setting of his own house. He is an older man with a COPD exacerbation, a man who pushes aside his walker and grabs his cane as he gets up, a man who provides a detailed summary of the sports he watched last week, a man whose medical record indicates that he served in the military. We bring him from the hospital to his apartment and set up the equipment, the band that goes on his arm to monitor his heart rate, and the electronic device that he can use to communicate with his nurse. He leans back in his chair in the living room. I turn to go for the day, thinking that I know what there is to learn about this patient. But then, he asks if I could stay for a few more minutes. He leans over and opens a large case on the floor, and he takes out a trumpet, his large hands carefully holding the instrument. The man plays a vibrant Jazz tune, my foot tapping along. When he sets the trumpet back in the case, I find myself sitting in a chair close by, listening while he tells me of how he came to be in this town. The patient tells me of his military career, tells me of the sports he participated in himself, tells me of his grandchildren who like to gather to hear him playing the trumpet when they come over.

I also think I know the older woman whose chart indicates that she likes dogs and cats and has difficulty being consistent with her medications. But then I come to see the patient at her house and am met by her husband by the entrance to their farm, a man with a beard and mud-splattered boots from many days of working on the farm. He leads me along a dirt path to their house, holding a boombox on one shoulder and a golf club in his grasp. The man looks around warily, increasing my own concern. I ask what he is worried about. He tells me of the rooster who defends the farm, the rooster who might consider me an intruder. I stay close to the man as we go to the patient’s house. There, I find her sitting on a chair in the living room and watching television. She’s holding a cat, and there are dogs all around her chair, cats and dogs all across the living room. I nearly trip on a dog as I walk into the house. Her chart did not mention what she tells me here, that she brings every dog and cat she finds on the street into her house where she feeds each one of them. My glance around the house tells me what she doesn’t, that she takes more care of her cats and dogs than of herself, with more food in their bowls than is found on her plate. I now appreciate the reason for her urinary tract infection that did not seem to initially improve with outpatient antibiotics prior to her coming to the hospital and needing IV antibiotics, since she admits that she has difficult consistently taking medications. She is busy helping her husband on the farm, busy caring for her dogs and cats, busy chasing the rooster who insists on pushing visitors out of the farm.

Then there is also an older woman whose apartment is filled with the smell of beans and rice when I walk in. She has bacteremia, with an antibiotic infusing as she hurries around the kitchen, her priority to prepare lunch for when her grandchildren come later today. I find myself more her assistant than her clinician, asking her medical questions while getting the ingredients she asks for, checking the cellulitis on her leg while she makes tacos and tells me that she has improved so much. In the hospital, the patient didn’t talk much with clinicians, pushing away the food that was brought to her and not wanting to go walking in the hallways. Here, I find it difficult to get a word in around her chatter, in which she tells about the manner in which she prepares tacos and how her approach is different from how tacos are often prepared. When I am about to go for the day, she approaches and tucks a taco wrapped in foil into my jacket. I tell her that this isn’t needed at all, even that I’m not allowed to take food from patients. But she insists, telling me that the people who determined those rules haven’t tried a taco like those that she prepares. She won’t allow me to leave without one of her tacos, and she stands on the porch to check that I get to my car without issues.

We think we get to know our patients in the hospital, that their charts and our interactions with them in their hospital room tell us what we need to know. But it is only when I started to meet patients in their own environments, when I stepped into spaces where each glance revealed more details of who my patients are, revealed what my patients care about, revealed the complexities of their experiences that their hospital room could not show me, that I felt I could really get to know my patients.

## Data Availability

To protect patient confidentiality, all identifying information has been removed or altered. The individuals described cannot be identified, and all details have been anonymized in accordance with ethical standards.

